# Reference Intervals for Trace Elements in Canine Plasma †

**DOI:** 10.3390/ani16020264

**Published:** 2026-01-15

**Authors:** Belén Larrán, Marta López-Alonso, Marta Miranda, María Luisa Suárez, Inmaculada Orjales

**Affiliations:** 1 Department of Animal Pathology, Faculty of Veterinary Medicine, Universidade de Santiago de Compostela, 27002 Lugo, Spain; belen.larran.franco@usc.es; 2 Department of Anatomy, Animal Production and Veterinary Clinical Sciences, Faculty of Veterinary Medicine, Universidade de Santiago de Compostela, 27002 Lugo, Spain; marta.miranda@usc.es (M.M.); maruska.suarez@usc.es (M.L.S.); inma.orjales@usc.es (I.O.)

**Keywords:** minerals, metals, toxic elements, heavy metals, canine, ICP-MS

## Abstract

Trace elements are essential for organisms, and their involvement in diverse diseases is increasingly recognised. Interest is increasing in veterinary medicine, particularly in relation to canine diseases. However, reference intervals for trace elements in dogs remain scarce. Plasma samples from 140 dogs were analysed by inductively coupled plasma mass spectrometry to determine the levels of 13 trace elements. Reference intervals (µg/L) were established for the following 12 elements: As, 0.417–8.17; Co, 0.039–1.33; Cr, 2.41–13.3; Cu, 296–790; Fe, 846–3643; Hg, 0.235–2.33; Ni, 0.567–9.04; Mn, 1.90–7.28; Mo, 1.43–12.7; Pb, 0.285–2.82; Se, 200–434; and Zn, 415–1095. However, Cd was below the limit of quantification in 77% of the samples. No differences in trace element concentrations were observed in relation to breed or reproductive status. Statistically significant differences were found in relation to sex (Cu, Mo, Zn), age (Co, Cu, Mo, Mn, Se, Zn), and size (Cu, Mo, Se, Zn); however, the magnitude of these effects varied among elements and was generally weak. Nevertheless, these factors should be considered when assessing trace element status. These reference intervals constitute an important resource for both clinical evaluation and future research.

## Simple Summary

Trace elements, or microminerals, are essential nutrients for all organisms, playing roles in structural functions and participating in numerous biochemical reactions. Both deficient and excess levels of these elements can be harmful, potentially leading to disease. Evidence from human studies shows that trace elements are implicated in multiple diseases, acting as preventive or predisposing factors and influencing the response to treatment and disease prognosis. For these reasons, trace element levels are increasingly studied in veterinary medicine, particularly in relation to diverse canine diseases. However, few studies have analysed large groups of healthy dogs, and reference intervals have not been established for most trace elements. In this study, 140 canine plasma samples were analysed with the aim of establishing the reference intervals for 13 trace elements. The samples were analysed by inductively coupled plasma mass spectrometry, which has a very low limit of detection for trace element measurement. The influence of biological factors on the variability in element levels was also assessed. The reference intervals established will be valuable for clinical evaluation and future research.

## 1. Introduction

Trace elements (also known as microminerals) are mineral elements present in the body in very small amounts, typically in the ppm or ppb range. Although only required in trace quantities, they are essential nutrients that must be obtained through the diet. These elements participate in a wide variety of physiological functions, e.g., as structural components or as cofactors in numerous enzyme reactions. Consequently, adequate trace element status is crucial for correct immune function, antioxidant defence, cognitive processes, growth and reproduction, among many other functions [1,2,3]. Both deficient and excess levels of essential elements can cause disease and disrupt multiple physiological functions. Likewise, exposure to toxic metals such as As, Cr, and Hg can compromise normal biological processes.

In addition to their capacity to cause diseases, trace elements are known to influence disease prevention, predisposition and prognosis and also treatment response across a wide range of conditions in human medicine [4,5,6]. Trace element status in dogs is an emerging research topic in veterinary medicine, and recent studies have investigated trace element concentrations in relation to conditions such as idiopathic epilepsy, pyometra, leishmaniasis, parvovirus infection and endocrine disorders [7,8,9,10,11,12]. Circulating concentrations of trace elements were determined in these studies, as this is a practical, minimally invasive approach to assessing the overall trace element status. However, comprehensive reference values in dogs remain scarce, and most studies include small control groups (≤20 dogs). Current guidelines published by the American Society for Veterinary Clinical Pathology (ASVCP) [13] recommend including a minimum of 120 individuals for calculation of reference intervals by nonparametric methods. To our knowledge, only two studies have assessed trace elements in cohorts larger than 70 dogs, by using spectrophotometric or atomic absorption methods to analyse serum samples [14,15]. In addition, the only reference interval reported was for Zn [14]. The analytical technique and type of sample used are important considerations in trace element studies. Atomic absorption spectrometry or spectrophotometry have been used in many studies in dogs. However, mass spectrometry techniques such as inductively coupled plasma mass spectrometry (ICP-MS) offer higher specificity and accuracy, as well as lower detection limits, which are particularly important for elements that occur at lower concentrations [1].

Due to the limited data available regarding multiple trace elements in large canine cohorts, the objective of this study was to determine plasma concentrations of thirteen trace elements, by using ICP-MS and following current ASVCP guidelines. Additionally, as a secondary exploratory aim, the influence of different biological factors (age, breed, sex, neuter status, weight) on the variability in element concentrations was also evaluated, hypothesising that these factors could exert measurable effects.

## 2. Materials and Methods

### 2.1. Sample Collection

This was a retrospective study and used plasma samples stored in a biobank. All samples consisted of remnants of plasma samples collected in heparin tubes (micro sample tube Lithium heparin LH, SARSTEDT, Sarstedtstraße, Nümbrecht, Germany) during routine clinical procedures, with dogs in a fasted state. Plasma was separated prior to storage and subsequently stored at −20 °C until analysis. Samples were thawed once only, immediately before acid digestion for ICP-MS determination. The blood samples were originally collected from dogs attending the Rof-Codina University Veterinary Hospital (Lugo, Spain) between January 2022 and July 2025, for health evaluation.

Eligibility criteria were as follows: (i) asymptomatic; (ii) older than 6 months; (iii) attending the hospital for routine procedures, such as health evaluation, vaccination or neutering; (iv) no abnormalities detected on clinical examination; (v) no clinically relevant abnormalities identified in laboratory analyses, including blood cell counts and a basic biochemistry panel; and (vi) primarily fed a non-prescription commercial dry diet and no additional vitamin or mineral supplementation. For dietary assessment, dogs were considered to consume a commercial dry diet if caregivers reported that more than 75% of the dog’s daily intake consisted of commercial dry feed. Information on the specific brands or treats consumed was not systematically recorded. The biochemistry panel included alanine aminotransferase, alkaline phosphatase, blood urea nitrogen, creatinine, albumin, globulin and glucose. Age, breed, sex, neuter status, weight, diet, complete blood counts and biochemical parameters were recorded.

Data collection followed Directive 2010/63/EU on the protection of animals used for scientific purposes (European Parliament, 2010), and the trial complied with Spanish legislation on animal care (Real Decreto 53/2013). All sampling methods and experimental procedures were approved by the Bioethics Committee of the Rof-Codina University Veterinary Hospital (protocol number AELU001/23/INVMED(02)/OUTROS(04)/MLSR/01).

### 2.2. Sample Preparation and ICP-MS Analysis for Determination of Trace Elements

Plasma samples were subjected to acid digestion before analysis by ICP-MS. Specifically, 0.4 mL of plasma was mixed with 0.3 mL concentrated HNO_3_ and 0.2 mL H_2_O_2_ in propylene tubes. The mixture was maintained at 60 °C in open vessels for at least 2 h to allow digestion of the samples. The resulting digest was diluted to 3 mL in ultrapure water. The diluted digest was centrifuged at 2000 rpm for 5 min, and the supernatant was collected for subsequent analysis of trace and toxic elements by ICP-MS. The concentrations of the following 13 elements were determined: arsenic (As), cadmium (Cd), cobalt (Co), chromium (Cr), copper (Cu), iron (Fe), mercury (Hg), manganese (Mn), molybdenum (Mo), nickel (Ni), lead (Pb), selenium (Se) and zinc (Zn).

Trace element analysis was conducted in a mass spectrometer (Agilent 7900 ICP-MS system, Agilent Technologies, Tokyo, Japan). The sample introduction system consisted of an autosampler, a double-pass spray chamber with Peltier system (Agilent Technologies, Tokyo, Japan), a glass concentric nebulizer (MicroMist low-flow nebulizer, Glass Expansion, West Melbourne, Australia) and a quartz torch (Agilent Technologies, Tokyo, Japan). The elemental concentrations were quantified using dedicated software (Agilent ICP-MS Mass Hunter 5.1, Version D.01.01, Agilent Technologies, Tokyo, Japan). The operational parameters were established as follows: plasma flow rate, 15 L/min; nebulizer flow rate, 1.1 L/min; sample depth, 8; sample flow rate at 0.1 rpm, plasma radiofrequency power at 1550 W, and spray chamber temperature maintained at 2 °C. Helium was used as the collision gas (4 mL/min) for all elements, while Se was measured using hydrogen in reaction mode (4.2 mL/min) to minimise spectral interferences. Elements were quantified using the following isotopes: 75As, 111Cd, 59Co, 52Cr, 63Cu, 56Fe, 202Hg, 55Mn, 95Mo, 60Ni, 208Pb, 78Se, and 66Zn. An internal standard of Rh was continuously introduced online to correct for instrumental drift and matrix effects. The analyses were carried out at the research infrastructures unit of the University of Santiago de Compostela (Lugo, Spain). This laboratory operates under a stringent analytical quality control system and holds ISO accreditation, ensuring the reliability and traceability of the result. Calibration curves (ranging from 0.2 to 10,000 μg/L) were constructed daily to ensure analytical accuracy. Calibration was performed using a multi-element standard solution, with element-specific concentration ranges selected according to the expected levels in the samples. For this purpose, fresh standard solutions were analysed before the plasma samples. The correlation coefficient values for the detection responses of the ICP-MS instrument were higher than 0.999 and the relative standard deviations were below 5%. All samples were analysed in triplicate. An analytical quality control procedure was used to verify the results (summarised in Table 1). Analytical blanks were included, and the limit of detection (LOD) and limit of quantification (LOQ) were calculated as 3 and 10 times the standard deviation of the blanks, respectively. The LOD values were sufficient for most elements, except Cd, Pb and Co. For Pb and Co, only 4 and 3 samples, respectively, were below the LOD and were assigned LOD/2 for statistical analysis. This approach was not applied to Cd, as the concentrations of this element were below the LOD in more than 15% of samples (only values above the LOQ were considered). The accuracy of the method was assessed by using canine plasma samples spiked in the laboratory with appropriate concentrations of the elements (up to 2–10 times higher than the normal levels in the samples) and a certified reference material (CRM; Seronorm™ Trace Elements Serum L-2, Billingstad, Norway). Overall, good recovery was achieved for the spiked serum samples and the certified elements in the CRM (Table 1). Recovery of elements not certified in the CRM (As, Cd, Mo, Pb) was only indicative. The accuracy of the determinations was considered acceptable. Analytical precision was evaluated using certified reference materials. Intra-run precision was determined by repeated measurements of the same certified reference sample within a single analytical batch, using ten replicates. Inter-run precision was evaluated by repeated analysis of the certified reference material across ten independent analytical runs on different days. Precision was expressed as the standard deviation divided by the mean, and both intra-run and inter-run measurements were within acceptable limits, being less than 10% for intra-run precision and less than 15% for inter-run precision, confirming the reproducibility of the ICP-MS measurements.

### 2.3. Data Analysis

Data normality was assessed using histograms and the Shapiro–Wilk test. Outliers were identified using Horn’s algorithm with Tukey’s interquartile fences, after prior transformation of non-normally distributed data. Values exceeding Q1 − 3 × IQR or Q3 + 3 × IQR (where IQR is the interquartile range) were considered extreme outliers. After visual inspection of the initial 152 samples, dogs with extreme outliers were found to typically present outlying values in two or more trace elements, suggesting a non-random deviation potentially related to preanalytical factors. Therefore, when two or more trace elements from the same dog were classified as extreme outliers, the entire subject was excluded from further analyses to avoid biassing reference interval estimation. After these exclusions, which resulted in the removal of 12 samples, no additional extreme outliers were detected in the trace element results of the remaining 140 dogs. Following the ASVCP guidelines [13], reference intervals were established using a non-parametric method with 90% confidence intervals for the reference limits, as the sample size exceeded 120.

Non-parametric tests were used to assess the impact of biological factors (age, sex, neuter status, weight, breed) on trace element concentrations (as in most cases the data were not normally distributed). The distributions of sex and neuter status across groups were first evaluated using the Chi-square test, and differences in trace element concentrations between categories were then assessed using the Mann–Whitney U test. Only breeds represented by three or more individuals were included in the analysis, and comparisons were conducted using the Kruskal–Wallis test. Age and weight were examined as continuous variables, using Spearman’s correlation, and as categorical variables after grouping, using the Kruskal–Wallis test. The dogs were categorised by life stage, as follows: puppies (6–12 months), young adults (>1–4 years), mature adults (>4–6 years) and pre-geriatric dogs (7–9 years). The body weight categories considered were small (<10 kg), medium (10–25 kg), large (>25–45 kg) and giant (>45 kg).

All statistical analyses were conducted using SPSS Statistics (version 29.0.1.0; IBM, Armonk, NY, USA). Reference intervals and graphical outputs were generated using Reference Value Advisor 2.1. [16], a freeware tool integrated in Microsoft Excel (ver. 2510 Microsoft, Redmond, WA, USA).

### 2.4. Animal Characteristics

After removal of outliers, 140 samples were analysed. A summary of the biological factors is presented in Table 2. Chi-square tests indicated no significant differences between sex and neuter status (*p* = 0.554). The mean ± SD age was 3.80 ± 2.65 years (range 0.5–9.96) and the mean body weight was 26.1 ± 13.2 kg (range 1.7–60.0 kg). Regarding breed, mixed-breed dogs were the most common (*n* = 86; 61%).

## 3. Results

### 3.1. Reference Intervals for Trace Elements Measured in Plasma

**Figure 1:**
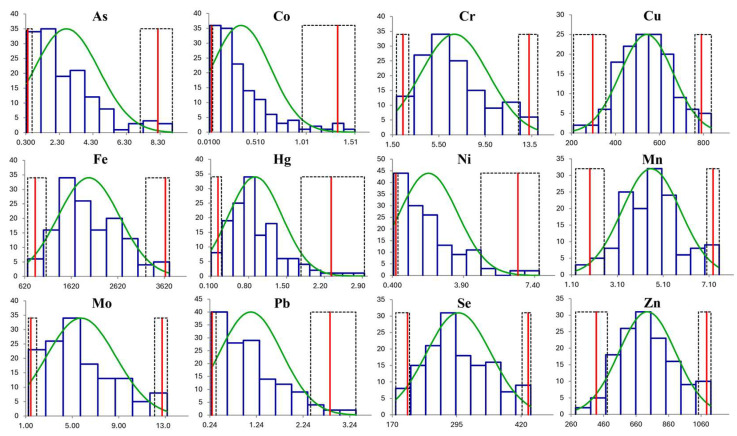
Distribution of plasma concentrations of 12 trace mineral elements in 140 healthy dogs. Observed concentration distributions are shown as dark blue histograms, and the fitted distributions are shown as green lines. The estimated reference limits are indicated by red lines, and their 90% confidence intervals are indicated by dashed black lines. The *X*-axis represents trace element values, and the *Y*-axis represents the number of observations. All mineral concentrations are expressed in µg/L. According to the Shapiro–Wilk test, only Cu, Mn, and Zn concentrations were normally distributed.

Reference intervals and descriptive statistics for 12 elements are presented in Table 3, and the corresponding distribution histograms are included in Figure 1. The Shapiro–Wilk test revealed that only the Cu, Mn and Zn concentrations were normally distribution. The concentrations of Cd were only above the LOQ (0.0325 µg/L) in 32 samples, and the mean concentration in these samples was very low (0.0871 ± 0.0513 µg/L). As 77% of values were below the LOQ, the reference interval was not calculated for Cd, and this element was excluded from further analyses.

### 3.2. Effect of Biological Factors on Trace Elements

Some of the biological factors considered (sex, age, weight) significantly affected the concentrations of Co, Cu, Mn, Mo, Se and Zn (Table 4).

Sex significantly affected Cu, Mo and Zn concentrations. Males had higher levels of Cu (*p* = 0.001; 11.0% difference) and Mo (*p* = 0.020; 17.1%), while females had higher levels of Zn (*p* = 0.043; 8.0%). Neuter status representation in the study was limited, with 112 entire and 28 neutered dogs. No significant differences were detected between neutered and intact animals for any of the elements evaluated.

Age was weakly positively correlated with levels of Cu (rho = 0.201, *p* = 0.017), Mo (rho = 0.203, *p* = 0.016), Mn (rho = 0.184, *p* = 0.030) and Se (rho = 0.275, *p* < 0.001) and negatively correlated with levels of Co (rho = –0.197, *p* = 0.020). The life stage categories significantly affected Co, Se and Zn concentrations. The Co levels decreased with age, with a significant difference between puppies and the pre-geriatric group (*p* = 0.043; 87.4%). Concentrations of both Se and Zn were higher in mature adults than in young adults (*p* = 0.011; 12.4% and *p* = 0.015; 15.0%, respectively). Although Mo levels were highest in the pre-geriatric group, the difference was not statistically significant, probably due to the high variability within groups (*p* = 0.095).

Body weight was weakly and positively correlated with concentrations of Cu (rho = 0.221, *p* = 0.009), Mo (rho = 0.293, *p* < 0.001), Se (rho = 0.256, *p* = 0.002) and Zn (rho = 0.206, *p* = 0.015). Weight category significantly affected the concentrations of Cu, Mo and Se. Large dogs had higher Cu levels than medium-size dogs (*p* = 0.004; 12.8%). Giant dogs had higher Se and Mo levels than medium-sized dogs (*p* = 0.012; 15.8% and *p* = 0.021; 34.1%, respectively).

Breed representation in the study was limited, as most of the dogs were mixed-breed. Only breeds represented by three or more individuals were included in the statistical analysis. No significant differences in trace element concentrations were detected among breeds. Manganese levels varied most across breeds (*p* = 0.019); however, none of the pairwise comparisons were significant after correction for multiple testing.

## 4. Discussion

### 4.1. Trace Element Reference Intervals

In this study, plasma concentrations of 13 trace elements were analysed in a cohort of 140 healthy dogs. Reference intervals were established for all of the elements except Cd, as the concentrations of this element were below the LOQ in most of the samples. Factors such as sex, age and weight were found to significantly (*p* < 0.05) affect the concentrations of elements such as Co, Cu, Mo, Mn, Se and Zn. However, the magnitude of these effects was generally small, and the minimal differences observed suggest substantial overlap between groups. Therefore, unified reference intervals are likely appropriate for interpreting trace element concentrations in this population. Comparison of the reference intervals with previously published data is restricted by the small number of studies that have included large cohorts of dogs. Furthermore, elements such as Fe, Cu, and Zn have been more frequently investigated in dogs, whereas others, such as Co, Mo and heavy metals have only been considered in a small number of studies.

Overall, the reference intervals calculated in the present study are consistent with those reported in two previous studies that also established reference intervals: one for Zn levels, measured in 197 dogs in the Czech Republic [14], and another for all of the elements, measured in 42 dogs in Spain, also by our research group [17]. For essential elements, the values obtained in the present study are also generally consistent with those reported by Puls (1994) [18] in a compilation of published data on mineral concentrations in different species. The main exception is Mn, for which Puls reported 20 µg/L [18], while we calculated a much lower interval, of 1.90–7.28 ug/L. This lower interval is consistent with findings of studies that included a healthy population [7,8,9,10] or just slightly lower that other reported levels, depending on the breed [15]. However, some studies have reported much higher Mn concentrations [19,20]. All of the aforementioned studies, except for [9], also by our research group, used serum samples, and therefore the differences between matrices are expected to be small, as demonstrated in other species [21,22]. Nonetheless, further research that directly compares trace element concentrations in plasma and serum samples is required in order to confirm this assumption. Clotting can influence trace element measurements, for example, by sequestering protein-bound elements during clot formation or by causing haemolysis. For example, lower Cu levels in serum than plasma in ruminants are attributed to the loss of ceruloplasmin during clot formation [21], and in humans, Zn can be released from platelets during clotting, increasing serum concentrations [22]. For other elements, such as Fe, even minor haemolysis, which occurs more frequently in serum, can result in increased measured concentrations [21,22].The analytical technique used is another important factor that should be considered when comparing studies. This is particularly important for elements present at very low concentrations, such as Co and heavy metals. For instance, the Co reference intervals calculated in the present study are consistent with those measured by ICP-MS in healthy dog populations [8,17], and they are similar to those reported in cattle [23] and humans [24]. However, in dogs, levels 4 to 10 times higher have been determined by AAS or ICP-OES [7,12,15]. Accurate analytical methods are also crucial for measuring toxic metals, as serum and plasma concentrations can be very low. Nonetheless, circulating levels are influenced by analytical sensitivity and also by environmental exposure, which can vary depending on the region where the animal lives. The intervals calculated for all of the heavy metals considered in this study are similar to concentrations previously reported for dogs [17] and for cattle and humans in the same study area (Galicia, Spain) [21,25]. In comparison with other countries, similar levels were found for Cr levels in dogs in the USA [26] and Turkey [7,12], while Ni levels are consistent with those reported in one study in Turkey [15] but lower than levels reported in another study the same country [12]. Conversely, the levels reported in the present study are much lower than those reported for Cd, Cr, Hg and Pb in the Republic of Korea [27] and for As, Cr, Ni and Pb in Poland [19].

### 4.2. Influence of Biological Factors

Although studies examining essential trace elements in healthy dogs in relation to biological factors are scarce, those that have assessed variables such as age, sex, weight, and breed are summarised in Table 5.

Considering the effects of sex on differences in trace elements concentrations revealed some statistically significant differences: slight differences for Cu and Zn, and moderate differences for Mo. Male dogs had higher levels of Cu (*p* = 0.001; 11.0% difference) and Mo (*p* = 0.020; 17.1%), whereas female dogs had higher levels of Zn (*p* = 0.043; 8.0%). Higher Zn levels were also observed in bitches in two previous studies [14,28], although these studies reported larger differences (23.5–34.3%, Table 5). However, one study did not detect any sex-related differences in Zn levels [19]. One study also found higher Cu levels in male dogs [29], while two others reported no sex-related differences in levels of this element [19,28]. No previous studies have analysed Mo concentrations in dogs in relation to biological factors. In humans, information is also limited, but circulating Mo levels also seem to be higher in males, although the differences are generally small and not always statistically significant [30,31,32]. Consistent with previous findings in dogs, we did not detect any differences in regard to levels of As, Cr, Hg, Ni, Pb [19,27], Fe [19,33,34], Mn [19] or Se [28,29]. Similarly, no differences were observed neuter status, as previously reported for Zn [14]. However, the proportion of neutered individuals in our study was small, which limits the statistical power to detect potential effects. Therefore, to better assess the influence of reproductive status on trace element levels, future studies should determine and analyse this factor.

Some age-related statistically significant differences were found for Co, Cu, Mo, Mn and Se and Zn levels. A weak positive association between age and Cu (*p* = 0.017; rho = 0.201) or Mn (*p* = 0.030; rho = 0.184) levels was identified, but no statistically significant differences were observed between age groups. Two previous studies reported no association between age and levels of Cu [19,35] or Mn [19], while another study noted that the levels decreased with age, although the magnitude of the variation was not reported [29]. A weak positive association was also observed between Se levels and age (*p* < 0.001; rho = 0.275), and differences between age groups were small (Table 4). By contrast, another study reported that ageing was associated with decreased Se concentrations, but again the magnitude of this effect was not specified [29]. Regarding Zn levels, we did not observe any correlation with age of the dogs, and although the difference between young adults and mature adults was significant, it was minimal (*p* = 0.015; 15.0% difference).

**Table 5:** Serum and plasma concentrations (µg/L) of essential trace elements in canine studies, in relation to age, sex and breed. Studies are listed in chronological order of publication.

Study	N	Group	Co	Cu	Fe	Mn	Se	Zn	Metric	Sample ^(Method)^
[34]	20	M			1660 ± 261				A	Plasma ^(4)^
	14	F			1690 ± 441					
[33] *	34	0.5 Y; M			1180 ± 370				A	Plasma ^(4)^
	29	0.5 Y; F			1240 ± 340					
	12	1 Y; M			2210 ± 400					
	12	1 Y, F			2110 ± 43					
[35]	27	0.5–5 Y		380 ± 20	1470 ± 100			380 ± 20	B	Plasma ^(3)^
	13	6–13.5 Y		370 ± 20	1180 ± 80			390 ± 30		
[29] *	21	6 Y		718 ± 32			236 ± 8		B	Plasma ^(2,4)^
	20	7 Y		877 ± 32			211 ± 8			
	18	8 Y		763 ± 19			224 ± 12			
	18	9 Y		610 ± 32			214 ± 10			
	13	10 Y		629 ± 19			220 ± 7			
[15]	12	Pointer	20 ± 2	860 ± 40	1530 ± 180	10 ± 4		760 ± 50	A	Serum ^(3)^
	6	Poodle	21 ± 3	770 ± 40	1280 ± 200	6 ± 5		770 ± 50		
	8	Setter	30 ± 3	780 ± 40	1180 ± 200	20 ± 4		720 ± 50		
	15	Lab.	34 ± 2	870 ± 40	1130 ± 200	9 ± 3		770 ± 50		
	8	GR	19 ± 3	870 ± 40	1510 ± 180	9 ± 4		710 ± 50		
	15	GSH	23 ± 2	890 ± 30	1320 ± 170	8 ± 3		760 ± 40		
	8	Malinois	22 ± 3	810 ± 40	1290 ± 180	9 ± 4		660 ± 50		
[19]	22	M		1342 ± 45	1783 ± 94	666 ± 43		1460 ± 58	B	Serum ^(2)^
	20	F		1419 ± 90	1491 ± 68	721 ± 73		1616 ± 90		
[36]	25	GH			1460 ± 104				B	Serum ^(4)^
	30	Others			1350 ± 95					
[37]	25	GH			1562 ± 519				A	Serum ^(4)^
	30	Others			1520 ± 524					
[38]	32	GH			1486 ± 522				A	Serum ^(4)^
	31	Others			1145 ± 263					
[39]	202	1–7.9 Y			1735				C	Serum ^(4)^
	186	8–11.9 Y			1455					
	97	>12 Y			1280					
[14]	99	M						582	C	Serum ^(4)^
	98	F						719		
[28]	8	M		540 ± 48			256 ± 25.7	700 ± 94	A	Serum ^(1)^
	6	F						940 ± 140		

Group: M = male; F = female; Y = years; Lab. = Labrador Retriever; GR = Golden Retriever; GSH = German Shepherd; GH = Greyhound. Metric: A = mean ± standard deviation; B = mean ± standard error; C = median. Method: ^(1)^ = inductively coupled plasma–mass spectrometry; ^(2)^ = inductively coupled plasma–optical emission spectrometry; ^(3)^ = atomic absorption spectrometry; ^(4)^ = spectrophotometry. * Only a subset of groups was included.

Similarly, three previous studies in dogs did not find any association between Zn levels and age [14,19,35]. Altogether, given the small effect sizes observed in correlations and group differences (Table 4), the observed age-related differences in Cu, Mn, Se and Zn levels in dogs seem unlikely to be of biological relevance.

No studies in dogs have specifically evaluated the effect of age on the concentrations of Co and Mo, and information on their metabolism of these elements in dogs is limited. Although related research in humans is also scarce, serum or blood levels of Mo and Co appear to remain relatively stable through life [25,30,40]. The weak negative correlation between age and Co levels (*p* = 0.020; rho = −0.197) observed in this study may be related to a decrease in intestinal absorption with age, as reported in rats and guinea pigs [41]; however, no such information is available for canines. Most data on Co levels in dogs are related to cobalamin (vitamin B12), of which Co is a component (this is the only well-established biological function of this element in non-ruminants). Similarly to Co, cobalamin levels have also been reported to decrease with age in dogs and cats [42,43]. However, it is not clear whether this could explain the observed negative correlation. Further research into Mo and Co metabolism in dogs is warranted.

Some studies have analysed the effect of age on Fe levels. We did not detect any significant age-related differences in our study population, consistent with most previous findings (Table 5). Likewise, no differences in dogs aged 1–4 years was reported in one study [34], and another found no variation between puppies (<1 year) and dogs older than 9 years [19]. By contrast, one study detected an increase in Fe concentrations in beagles between 6 and 10 months of age [33]. In another study, no differences were observed in adult or senior dogs, but Fe levels were significantly lower in the geriatric group (>12 years), and the study authors suggested that the lower Fe levels in these elderly dogs may be related to inflammation or unrecognised gastrointestinal blood loss [39]. However, the present study did not include dogs older than 10 years, and therefore age-related changes in Fe or other trace elements in truly geriatric dogs could not be assessed. Other researchers also noted a non-significant decline in an older group of dogs (6–13.5 years), possibly due to a smaller number of truly geriatric animals [35]. Overall, Fe concentrations appear relatively stable throughout the lifespan of dogs, with a potential decline occurring advanced geriatric ages, where further research is needed.

This study did not find any association between age and heavy metal concentrations, in line with two previous studies in dogs [19,27]. In one study, it was observed that serum concentrations of Cd, Hg, Pb and Cr tended to increase with age of dogs, but the differences were not statistically significant [27]. Plasma may not be the optimal matrix for assessing cumulative heavy metal exposure over a lifetime, as it primarily reflects recent exposure or the circulating fraction, whereas organs such as the liver or kidney provide a more accurate measure of long-term accumulation [44]. In addition, repeatability and accuracy at low concentrations can be limited for certain toxic metals. Nevertheless, plasma analysis could be sufficient to evaluate current exposure levels, as higher concentrations of heavy metals have been reported in other studies [19,27]. In addition, higher serum heavy metal concentrations were detected in dogs from industrialised areas than in dogs from rural areas [27]. Overall, plasma reference intervals should be considered a reference point rather than a substitute for organ-based assessment during long-term exposure.

Statistically significant differences related to body weight were also observed in this study, mainly because large and giant dogs had higher concentrations of Cu (*p* = 0.004; 12.8%), Se (*p* = 0.012; 15.8%), and Mo (*p* = 0.021; 34.1%) than medium-sized dogs. While the differences in Cu and Se were relatively small, the Mo levels were 15–39% higher in large and giant dogs than in small and medium-sized individuals. Only one study has examined weight as a factor and reported significantly higher Cu levels in small dogs than in large dogs [19], in contrast to the findings of the present study. One limitation of the present study is that the body condition score was not available for all dogs and was thus not included in the analyses. Research on body condition in healthy dogs is limited. One study found no impact on Zn levels [14], while comparisons between obese and normal body condition reported higher Fe concentrations in obese dogs and cats [12,45], although values remained within the reference interval established in the present study. Future studies should include body condition score as a factor in the analyses.

Although weight is partly associated with breed, no differences in trace element concentrations were detected in relation to breed. However, the effect of breed was not able to be reliably established in this study, as the representation of different breeds was low (see Section 3.1), with mongrel dogs accounting for 61% of the sample. This restricts the applicability of the reference intervals to purebred populations and limited the ability to perform robust breed-specific analyses. Some research has been conducted in specific breeds, although sample sizes were also limited. For example, a study including seven different breeds found significant differences in levels of Cu, Mn, Ni, Cd, and Co, although the variations were reported to be small [15] (Table 5). Other studies have investigated Fe levels in greyhounds, which are known to have higher haematocrit and haemoglobin levels than other breeds. Two studies found no differences in Fe levels relative to other breeds [36,37], while one reported that Fe levels were 26% higher in greyhounds than in other breeds (Table 5) [38]. Overall, even though breed-related differences in plasma trace elements are thought to be minor, they have not yet been thoroughly investigated in veterinary medicine. Diet is another important factor to consider in trace element studies, as it represents the primary source of the elements. In this study, only dogs consuming predominantly dry food were included. The dogs were therefore expected to receive adequate amounts of essential elements as commercial dry dog food is routinely supplemented with trace elements in order to meet legal requirements. Nonetheless, the diet could have been assessed in greater detail, and this is recommended for future research. Some studies have evaluated the effect of diet, but conflicting results have been obtained [14,19,27], highlighting the need for further investigation.

### 4.3. Limitations

This study has several limitations that should be considered when interpreting the results. The reference intervals reported here apply specifically to heparinized plasma analysed by ICP-MS and should not be directly extrapolated to other sample types. Cadmium concentrations were below LOQ for most samples; therefore, no reference interval could be established, and Cd values should be interpreted descriptively only, not used for clinical decision-making. Also, although only asymptomatic dogs were included, subclinical inflammation or micronutrient imbalances cannot be ruled out and may contribute to biological variability. The representation of purebred and neutered dogs was limited due to the small number of samples, and no dogs older than 10 years were included, restricting interpretation for geriatric populations. Additionally, a complete nutritional assessment and body condition score were not comprehensively recorded. Finally, as this was an observational study, the mechanisms underlying the observed effects of biological factors cannot be determined. The study focused on reporting patterns in trace element concentrations, highlighting the need for future research to investigate the physiological and metabolic mechanisms behind these differences.

## 5. Conclusions

Following ASVCP guidelines, plasma samples of 140 healthy dogs were analysed by ICP-MS, and reference intervals were calculated for 12 trace elements. In 77% of the samples, the Cd concentrations were below the limit of quantification, and a reference interval was therefore not calculated for this element. The reference intervals established constitute an important resource for both clinical evaluation and future research.

Trace element concentrations were significantly (*p* < 0.05) affected by the factors sex (Cu, Mo, Zn), age (Co, Cu, Mo, Mn, Se, Zn), and size (Cu, Mo, Se, Zn). However, the magnitude of these effects varied among elements, was generally small, and may not be clinically meaningful in individual dogs. For this reason, unified reference intervals are considered appropriate for interpreting trace element concentrations in this population. Nonetheless, these biological factors should be considered when evaluating trace element status and designing future studies. Although there were no differences in trace element concentrations in relation to breed or reproductive status, the limited representation of certain breeds and neutered dogs reduces the power to detect potential effects, further research is needed to clarify the influence of these factors.

## Figures and Tables

**Table 1:** Results of the analytical quality programme, expressed as mean ± standard deviation (SD), used to determine the trace element concentrations in this study.

Element	LOD (Mean; μg/L)	LOQ (Mean; μg/L)	Spiked Samples	Certified Reference Material	
			Recovery (mean ± SD; %)	Certified Value (Mean; μg/L)	Recovery (Mean ± SD; %)
As	0.0119	0.0396	110 ± 5.9	(0.21)	166 ± 34.5
Cd	0.00975	0.0325	100 ± 4.8	(0.09)	152 ± 73.1
Co	0.00378	0.0126	105 ± 6.2	3.03	106 ± 6.52
Cr	0.0260	0.0867	110 ± 12	4.96	116 ± 20
Cu	0.0188	0.0627	100 ± 3.4	1961	100 ± 11.4
Fe	0.0999	0.333	101 ± 4.6	1910	106 ± 9.20
Hg	0.0140	0.0467	103 ± 7.3	1.99	113 ± 13.8
Mn	0.0282	0.0941	107 ± 6.0	14.8	108 ± 8.06
Mo	0.00590	0.0196	105 ± 4.1	(0.64)	139 ± 29.7
Ni	0.0174	0.0578	101 ± 7.7	9.7	107 ± 8.65
Pb	0.00468	0.0156	108 ± 7.1	(0.69)	207 ± 118
Se	0.0550	0.183	101 ± 5.2	136	106 ± 8.55
Zn	0.091	0.303	104 ± 5.1	1940	107 ± 8.65

Values in parentheses are indicative CRM values (not certified) used to calculate recoveries. LOD = limit of detection; LOQ = limit of quantification. Reported LOD and LOQ are pre-dilution values.

**Table 2:** Biological characteristics of the 140 dogs included in the study.

Biological Factor	Category	*n*
Sex	Female	73
	Male	67
Neuter status	Entire	112
	Neutered	28
Age	Puppies (6–12 m)	17
	Young adults (>1–4 y)	69
	Mature adults (>4–6 y)	31
	Pre-geriatric (7–9 y)	23
Size	Small (<10 kg)	15
	Medium (10–25 kg)	57
	Large (>25–45 kg)	53
	Giant (>45 kg)	15
Breed: Mixed-breed (*n* = 86), Labrador Retriever (*n* = 6), German Shepherd (*n* = 6), Beagle (*n* = 5), Golden Retriever (*n* = 3), Boxer (*n* = 3), Border Collie (*n* = 3), Can de Palleiro (Galician Shepherd) (*n* = 3), Spanish Mastiff (*n* = 3), English Setter (*n* = 3), Spanish Hound (*n* = 3); other breeds with <3 individuals (*n* = 16).		

m = months; y = years.

**Table 3:** Reference intervals and descriptive statistics for plasma concentrations of 12 trace mineral elements in 140 healthy dogs. All results are expressed in µg/L.

	Reference Interval	90% CI Lower Limit	90% CI Upper Limit	Mean ± SD	Median	Range
As	0.417–8.17	0.340–0.680	7.12–9.03	2.72 ± 1.94	2.09	0.340–9.03
Co	0.0390–1.33	0.0200–0.0500	0.969–1.51	0.342 ± 0.303	0.245	0.02–1.51
Cr	2.41–13.3	1.85–2.91	12.4–14.0	6.84 ± 2.93	6.13	1.85–14.0
Cu	296–790	209–356	761–835	542 ± 121	538	209–835
Fe	846–3643	683–1080	3223–3730	1999 ± 664	1907	683–3730
Hg	0.235–2.33	0.110–0.300	1.77–2.94	0.923 ± 0.496	0.825	0.110–2.94
Ni	0.567–9.04	0.460–0.675	5.25–11.1	2.18 ± 1.44	1.83	0.46–7.62
Mn	1.90–7.28	1.28–2.52	7.01–7.55	4.58 ± 1.28	4.60	1.28–7.55
Mo	1.43–12.7	1.22–1.94	12.1–13.2	5.76 ± 2.99	5.31	1.22–13.2
Pb	0.285–2.82	0.270–0.375	2.40–3.37	1.12 ± 0.671	0.960	0.270–3.37
Se	200–434	177–204	422–439	299 ± 62.4	287	177–439
Zn	415–1095	288–483	1044–1121	729 ± 175	719	288–1121

CI = confidence interval; SD = standard deviation.

**Table 4:** Mean ± standard deviation of the effects of biological factors on trace element concentrations (µg/L).

Variable		Co	Cu	Mn	Mo	Se	Zn
Sex	M (67)	0.333 ± 0.306	573 ± 108	4.55 ± 1.17	6.27 ± 2.94	294 ± 59.4	699 ± 177
	F (73)	0.349 ± 0.302	513 ± 125	4.61 ± 1.38	5.28 ± 2.98	303 ± 65.2	757 ± 169
	*p*	0.801	0.001 *	0.731	0.020 *	0.572	0.043 *
Age	P (17)	0.605 ± 0.433 ^a^	529 ± 132	4.56 ± 1.47	5.17 ± 2.62	279 ± 68.0 ^ab^	771 ± 165 ^ab^
	YA (69)	0.322 ± 0.280 ^ab^	524 ± 123	4.36 ± 1.24	5.53 ± 3.14	287 ± 58.5 ^a^	686 ± 171 ^a^
	MA (31)	0.318 ± 0.288 ^ab^	566 ± 122	5.00 ± 1.07	5.51 ± 2.51	325 ± 60.7 ^b^	797 ± 169 ^b^
	PG (23)	0.237 ± 0.142 ^b^	573 ± 96.6	4.66 ± 1.42	7.19 ± 3.14	313 ± 61.2 ^ab^	734 ± 174 ^ab^
	*p*	0.043 *	0.251	0.101	0.095	0.011 *	0.015 *
Size	S (15)	0.365 ± 0.211	574 ± 119 ^ab^	4.69 ± 0.840	4.99 ± 2.40 ^ab^	308 ± 58.2 ^ab^	748 ± 149
	M (57)	0.328 ± 0.355	499 ± 114 ^a^	4.29 ± 1.31	5.23 ± 3.17 ^a^	279 ± 55.0 ^a^	692 ± 192
	L (53)	0.339 ± 0.288	567 ± 120 ^b^	4.77 ± 1.23	6.08 ± 2.87 ^ab^	309 ± 67.8 ^ab^	745 ± 170
	G (15)	0.378 ± 0.227	583 ± 108 ^ab^	4.85 ± 1.57	7.38 ± 2.71 ^b^	327 ± 56.2 ^b^	794 ± 120
	*p*	0.181	0.004 *	0.074	0.021 *	0.012 *	0.051

Numbers in brackets indicate the number of dogs in each group. Asterisks denote statistical significance at *p* < 0.05. Different superscript letters within a column and variable indicate statistically significant differences (*p* < 0.05). Sex: M = male; F = female. Age: P = puppy; YA = young adult; MA = mature adult; PG = pre-geriatric. Size: S = small; M = medium; L = large; G = giant.

## Data Availability

The data presented in this study are openly available in Zenodo at https://doi.org/10.5281/zenodo.17950939 (accessed on 16 December 2025), reference number 17950939.

## Abbreviations

The following abbreviations are used in this manuscript:

ASVCP	American Society for Veterinary Clinical Pathology
CRM	Certified reference material
ICP-MS	Inductively coupled plasma mass spectrometry
IQR	Interquartile range
LOD	Limit of detection
LOQ	Limit of quantification
